# Neuromuscular characteristics during weight loss in a professional boxer: a case study

**DOI:** 10.1152/jn.00307.2022

**Published:** 2022-09-21

**Authors:** Tetsuya Hirono, Saeko Ueda, Yukiko Mita, Kohei Watanabe

**Affiliations:** ^1^Laboratory of Neuromuscular Biomechanics, School of Health and Sport Science, Chukyo University, Toyota, Japan; ^2^Research Fellow, Japan Society for the Promotion of Science, Tokyo, Japan; ^3^Department of Human Nutrition, School of Life Studies, Sugiyama Jogakuen University, Nagoya, Japan

**Keywords:** blood test, boxing, high-density surface electromyography, motor unit, weight loss

## Abstract

It is important to understand the effects of rapid changes in weight on neuromuscular functions of combat athletes. The purpose of this case study was to investigate time-course changes in muscle strength, muscle size, and neural input during rapid weight loss in a professional boxer. One professional male boxer (26 yr) participated in two matches during measurements: welterweight (66.6 kg; weight loss: WL) and super welterweight (69.85 kg; control: CON). His muscle contraction properties and body composition were measured from 6 wk (baseline) before the matches to 1 wk after them. Maximal voluntary isometric knee extension torque, muscle cross-sectional area (mCSA) of the vastus lateralis using ultrasound, and high-density surface electromyography of the vastus lateralis during submaximal ramp-up contraction were measured. Individual motor units were identified, and modified discharge rates were calculated from a regression line between the recruitment threshold and discharge rates at 60%–70% of maximum torque according to the baseline value. His body weights for WL and CON decreased from 70.80 and 71.42 kg at the baseline to 68.75 and 71.36 kg immediately before the matches, respectively. Muscle strength changed little for either match. For WL, skeletal muscle mass and mCSA decreased, but there was no decrease for CON. The modified motor unit discharge rate for WL increased immediately before the match compared with other periods but did not change for CON. After rapid weight loss, neural input increased to compensate for lost muscle mass, and muscle strength was maintained.

**NEW & NOTEWORTHY** This case study found that neural input to muscle, which was evaluated by high-density surface electrocardiography, increased to compensate for the decline of body weight and muscle mass and to maintain muscle strength during rapid weight loss, while neuromuscular characteristics were not markedly changed during no significant weight loss.

## Graphical abstract

**Figure:**
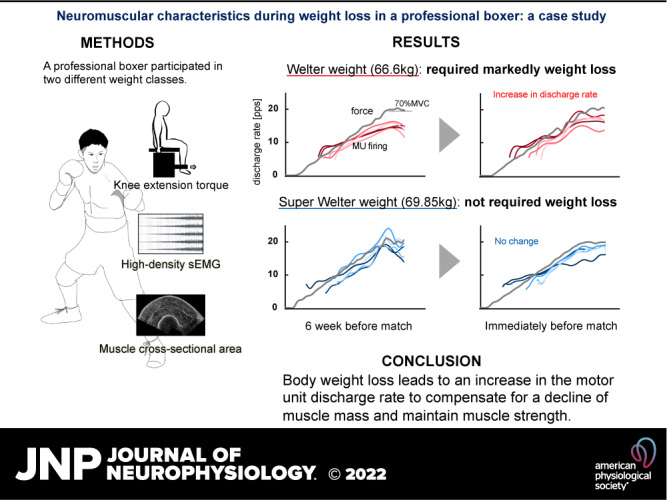

## BACKGROUND

Weight control is important for many athletes. Especially, participants of a weight class-based sport such as combat sports or weight lifting, need to lose body weight rapidly to drop below a set level. Rapid weight change can cause many physiological changes (1, 2) and alter performance (3). It is important to examine how rapid weight loss causes physiological effects and influences performance.

Focusing on effects of combining nutrition restriction and resistance training, a systematic review (4) showed that an energy-deficient state impairs lean mass gains as a result of resistance training, but muscle strength gain shows no difference between an energy-deficient state with resistance training and a control condition. These findings suggest that energy deficiency induces skeletal muscle decrease but does not influence muscle strength. Considering that muscle strength is determined by both morphological and neural factors, it is hypothesized that neural factors would compensate for a decrease in muscle mass to maintain muscle-exerting strength.

The purpose of this case study was to investigate neuromuscular characteristics in a professional boxer during rapid weight loss or nonsignificant weight loss before and after matches.

## METHODS

One professional boxer (26 yr old) in Japan participated in this case study. He was a welterweight fighter and ranked nationally. He was healthy and had no history of neuromuscular disorders or surgery. The purpose and procedures were explained to him before he provided informed written consent to participate in the case study. This study was conducted in accordance with the Declaration of Helsinki and approved by the Research Ethics Committee of Chukyo University (2021-056).

He participated in two matches during measurements: welterweight (66.6 kg; weight loss condition: WL) and super welterweight (69.85 kg; control condition: CON). The interval between the two matches was 4 mo. His body composition was measured every weekday. The maximum strength, muscle cross-sectional area, high-density surface electromyography (HDsEMG), and blood data were measured at a midpoint in each week. Data on the body composition were used as average values among weeks. The measurements were performed at 44–48 days (Pre6wk), 23–27 days (Pre3wk), 16–20 days (Pre2wk), 9–13 days (Pre1wk), and 2–6 days (Pre0wk) before and 1–5 days (Post1wk) after the matches.

Body weight, skeletal muscle mass, body fat, and total body water were measured using a weighing device employing bioelectrical impedance spectroscopy (InBody 430; InBody Japan, Tokyo, Japan).

A commercially available kit (DEMECAL; FUJIFILM Medical Co., Ltd., Japan) was used for blood data collection, and 0.065-mL samples of blood were obtained from a fingertip by the subject himself immediately after he woke up in the morning. The fingertip was punctured using an instrument (Naturalet EZ device; Arkray) and a disposable puncture needle (Naturalet EZ; Arkray). The variances that the kit can measure are listed in Table 1.

**Table 1.** Changes in blood test variances

	Pre6wk	Pre3wk	Pre2wk	Pre1wk	Pre0wk	Post1wk
Total protein, g/dL
WL	6.9	6.8	6.9	6.8	6.9	6.9
CON	6.9	7.1	7.2	7.1	7.1	7.1
Albumin, g/dL
WL	4.8	4.8	4.8	4.8	4.8	4.8
CON	4.8	4.7	4.6	4.7	4.7	4.7
AST (GOT), U/L
WL	34	28	27	28	26	29
CON	28	26	25	25	29	28
ALT (GPT), U/L
WL	33	23	18	19	21	24
CON	23	17	16	20	25	27
Total cholesterol, mg/dL
WL	162	143	146	163	154	192
CON	169	179	174	154	160	163
Glycerides, mg/dL
WL	63	60	41	36	89	96
CON	68	138	80	74	61	82
HDL-cholesterol, mg/dL
WL	71	64	63	69	76	79
CON	64	51	57	59	62	96
Uric acid, mg/dL
WL	6.1	5.9	5.5	5.5	5.5	4.6
CON	5.7	5	4.8	4.9	4.6	4.9
Urea nitrogen, mg/dL
WL	19	15.5	15.7	17.5	15.5	14.3
CON	18.4	12.9	14.7	14.5	14	15.9
Creatinine, mg/dL
WL	0.94	0.81	0.84	0.78	0.78	0.75
CON	0.75	0.79	0.76	0.74	0.74	0.74
Blood glucose level, mg/dL
WL	98	98	86	93	102	113
CON	93	119	102	85	95	103
HbA1c(NGSP), %
WL	5.2	5.3	5.3	5.3	5.3	5.4
CON	5.2	5.3	5.4	5.3	5.2	5.3

ALT, alanine aminotransferase; AST, aspartate aminotransferase; CON, the condition in the super welterweight match, which did not require significant body weight loss; HbA1c, hemoglobin A1c; HDL, high-density lipoprotein; NGSP, National Glycohemoglobin Standardization Program; WL, the condition in the welterweight match, which required rapid body weight loss.

Muscle cross-sectional area of the vastus lateralis was measured using an ultrasound device (LOGIQ e Premium, GE Healthcare) with a 10-MHz linear array probe. The muscle cross-sectional area of the vastus lateralis was determined from extended-field-of-view ultrasound images, following previous studies (5, 6). Transversal images were taken at three sites: 30, 50, and 70% of the distance from the greater trochanter to lateral condyle of the femur, determined as proximal, middle, and distal sites, respectively (7). The cross-sectional area was determined as the area surrounded by the fascia on the image. Images were taken twice each period. Averaged values for the three locations were used as the results for each period.

The maximum voluntary isometric knee extension torque was measured using a custom-made dynamometer (Takei Scientific Instruments Co., Ltd.) fixed to a force transducer (LU-100KSE; Kyowa Electronic Instruments). He was seated in it, the hip was flexed at 90°, and the knee was also flexed at 90°. Measurements of maximum strength were performed twice. The peak force during the contraction was recorded and the greater value of the two measurements was determined as the maximum voluntary contraction (MVC) force. The MVC torque was calculated by multiplying the MVC force and arm length, determined as the distance between the knee joint axis and force transducer.

**Figure 1.**
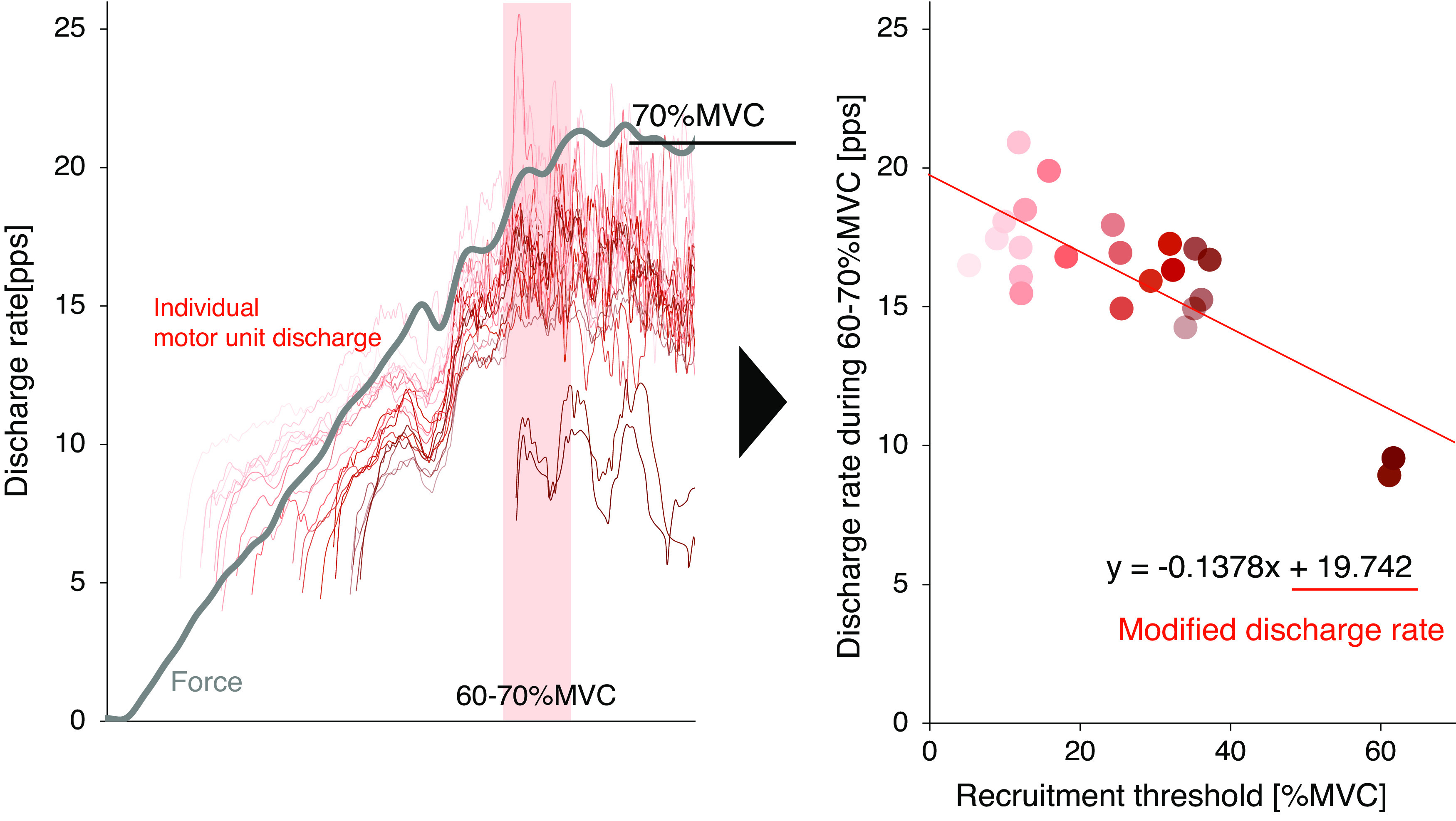
Measurements of individual motor unit discharge rate and calculation for the regression line between the recruitment threshold and discharge rate at 60%–70% of MVC. The *y*-intercept of the regression line was used as the modified discharge rate. MVC, maximum voluntary contraction.

After measuring MVC, he performed submaximal ramp-up contraction. According to our previous study (8), HDsEMG signals were recorded during ramp-up contraction to 70% of MVC (ramp-up rate: 5% per second), which was based on the MVC value at the baseline (Pre6wk) under each condition. To identify each motor unit recruitment threshold and further promote the detection of motor units, the ramp-up task was used. Details of the method were described in a previous study (8). HDsEMG signals were recorded from the vastus lateralis using a semidisposable adhesive grid of 64 electrodes (GR08MM1305, OT Bioelettronica, Torino, Italy). Electrodes were attached at the midpoint of the line between the head of the greater trochanter and inferior lateral edge of the patella. Monopolar surface EMG signals were filtered with a bandpass filter from 10 to 500 Hz and amplified by a factor of 256, sampled at 2,000 Hz, and converted to digital form (Sessantaquattro, OT Bioelettronica, Torino, Italy). The signal from the force transducer was synchronized with HDsEMG signals using this converter. The exerted and target forces were shown on a monitor in real time as visual feedback. Recorded monopolar surface EMG signals were transferred to analysis software (MATLAB R2019a, MathWorks GK, Tokyo, Japan), and individual motor units were identified by the convolution kernel compensation (CKC) technique using DEMUSE (ver. 5.0.1; The University of Maribor, Slovenia) (9, 10). Any physiologically irregular motor unit discharge (less than 4 and over 30 Hz, pulse-to-noise ratio less than 30 dB, and coefficient of variation of over 30%) was discarded according to our previous studies (8, 11). The recruitment threshold of individual motor units was defined at the force level where the first discharge was identified. The individual motor unit discharge rate was calculated as the mean value between 60% and 70% of MVC exerted, because the peak discharge rate could be ascertained with this duration. Scatter plots of the discharge rates and recruitment thresholds of individual motor units were analyzed, and the linear regression was calculated. The *y*-intercept of the regression line was defined as a modified discharge rate. The modified discharge rate is an indicator that considers individual motor unit recruitment thresholds. Thus, it was used for further analysis to examine the amount of neural excitability (Fig. 1).

To investigate the changes in the amount of neural excitability and anthropometric parameters, the modified discharge rate and, body weight or muscle cross-sectional area from six periods for each match were analyzed with the Pearson correlation coefficient. Statistical analyses were performed using SPSS version 25 (IBM Japan, Inc., Tokyo, Japan).

## RESULTS

**Figure 2.**
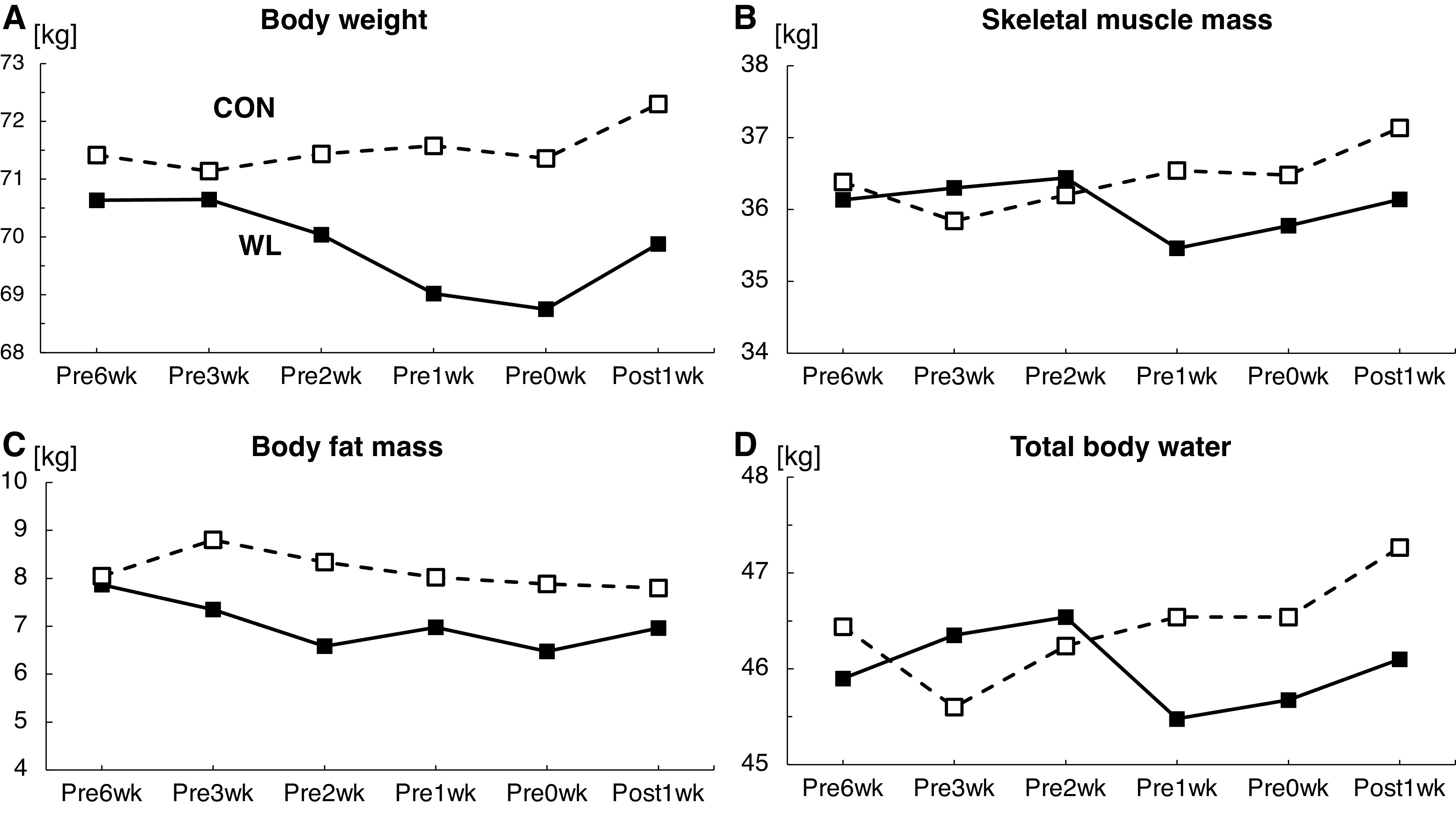
Changes in body weight (*A*), skeletal muscle mass (*B*), body fat (*C*), and total body water (*D*) measured using bioimpedance analysis. The black square and solid line represent the condition in the welterweight match (weight loss; WL), which required rapid body weight loss, and the white square and dotted line represent the condition in the super welterweight match (CON), which did not require significant weight loss.

His baseline body weights were 70.80 and 71.42 kg for welterweight (WL) and super welterweight (CON) matches, respectively. At Pre0wk, his body weight decreased to 68.75 and 71.36 kg for WL and CON, respectively (Fig. 2*A*). For WL, skeletal muscle mass decreased markedly from the baseline to match (36.1 to 35.8 kg). On the other hand, for CON, skeletal muscle mass changed little (36.4 to 36.5 kg) (Fig. 2*B*). The results of body fat mass and total body water are shown in Fig. 2, *C* and *D*.

**Figure 3.**
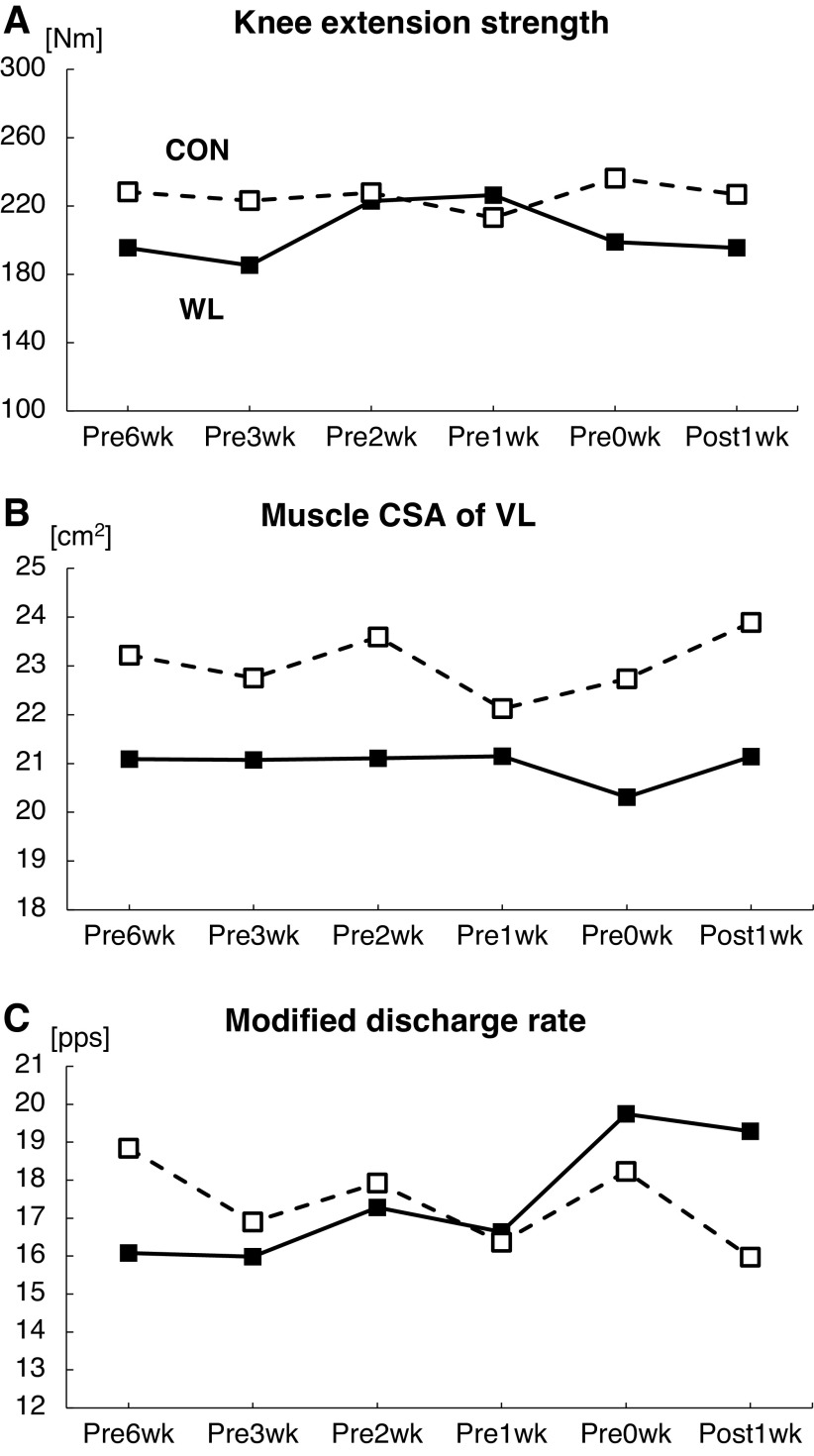
Changes in knee extension maximum voluntary isometric strength (*A*), muscle cross-sectional area of vastus lateralis (*B*), and modified motor unit discharge rate (*C*). The black square and solid line represent the condition in the welterweight match (weight loss; WL), which required rapid body weight loss, and the white square and dotted line represent the condition in the super welterweight match (CON), which did not require significant body weight loss. CSA, cross-sectional area; MU, motor unit; VL, vastus lateralis.

**Figure 4.**
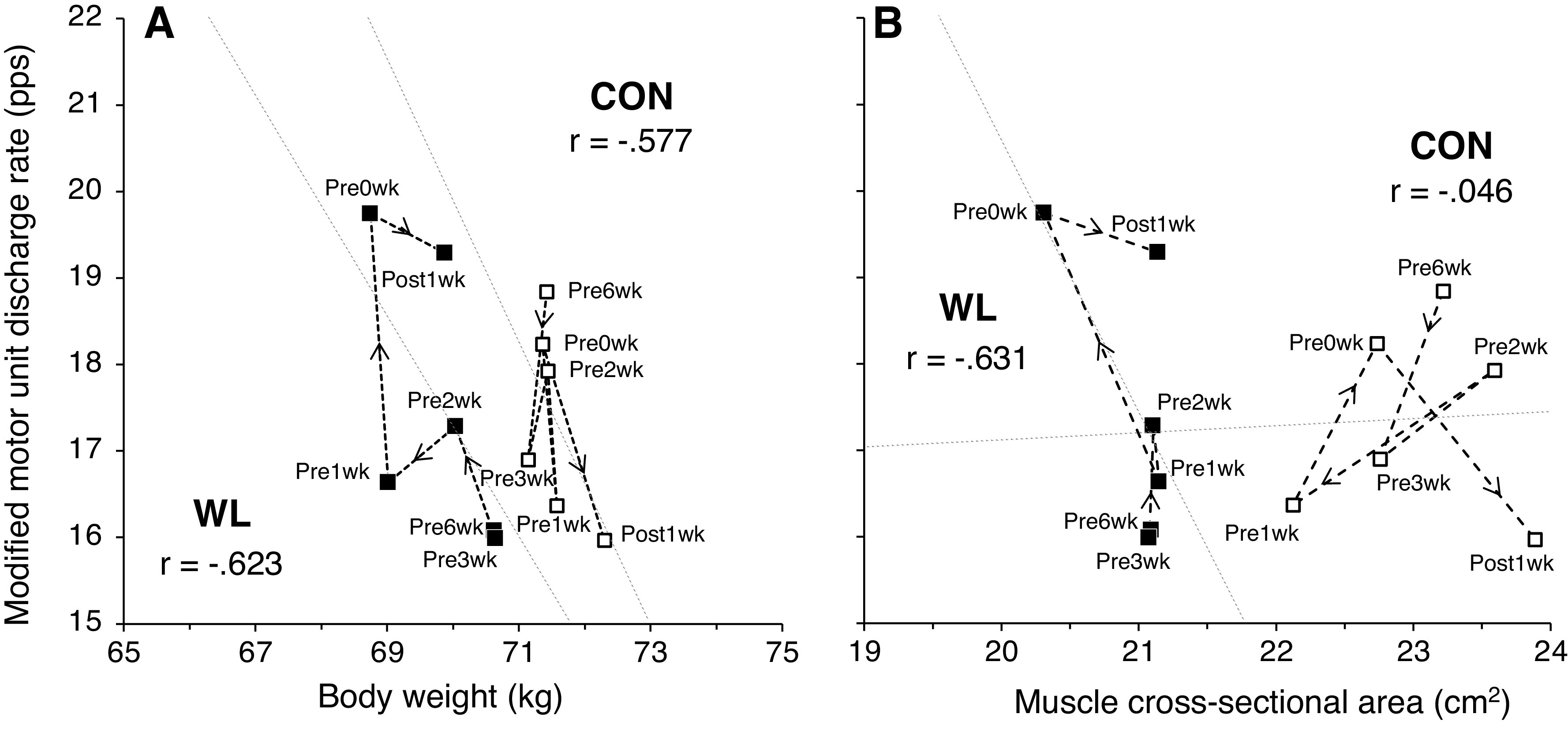
Association between motor unit discharge rate and body weight (*A*) or muscle cross-sectional area (*B*) under each condition. Black squares show the welterweight match (weight loss; WL), which required rapid body weight loss, and the white squares show the condition in the super welterweight match (CON), which did not require significant body weight loss.

The changes in muscle properties are presented in Fig. 3. Although muscle strength fluctuated during the periods, no changes accompanying weight loss were observed (Fig. 3*A*). For WL, the muscle cross-sectional area decreased markedly from the baseline to match (21.1 to 20.3 cm^2^). For CON, the muscle cross-sectional area did not decrease from the baseline to match (23.2 to 22.7 cm^2^) compared with WL (Fig. 3*B*). The number of detected motor units was 15.7 ± 5.2 in each section. The modified motor unit discharge rate was increased to the match for WL, but it was not increased for CON (Fig. 3*C*). Correlation coefficients between the motor unit discharge rate and body weight in six periods for WL and CON were −0.623 (*P* = 0.186) and −0.577 (*P* = 0.230), respectively (Fig. 4*A*). Correlation coefficients between the discharge rate and muscle cross-sectional area for WL and CON were −0.631 (*P* = 0.179) and −0.046 (*P* = 0.930), respectively (Fig. 4*B*).

## DISCUSSION

The present case study examined the changes in neuromuscular characteristics with body weight in a professional boxer under two conditions: one involved rapid weight loss, and the other involved no significant weight loss. The interval between the two matches was 4 mo, and so we believe that his performance levels changed little. In addition, blood sample tests revealed that all variances were in the standard range (Table 1). The weight loss was not pathological and may have represented an acute decrease in body water (1). The body weight and skeletal muscle mass reverted to the baseline values after the matches without pathological responses (Fig. 2, Table 1).

With his body weight loss, his skeletal muscle mass also reduced (Fig. 2*B*). Most body weight loss may be due to a decrease in muscle mass. A previous cross-sectional study reported that the motor unit and its size, which were estimated using the motor unit number index technique, were not different between healthy older adults and those with sarcopenia, but the whole muscle size differed (12). Although the previous work (12) was not a longitudinal study, it suggested that the decrease in muscle mass was not accompanied by motor neuron decrease. Considering aging with motor neuron plasticity, an initial increase in the motor unit size compensates for muscle atrophy, followed by a decline in the motor unit number (13, 14). In the present study investigating short-term changes in a young athlete, neural plasticity also played a role to compensate for the decrease in muscle mass because muscle strength did not decline but the motor unit discharge rate significantly increased accompanying the reduction of the muscle cross-sectional area (Fig. 3). Acute weight loss could cause neural input to increase as a mechanism to compensate for muscle mass decline. In Fig. 4, moderate correlation coefficients were noted between the motor unit discharge rate and body weight changes (Fig. 4*A*) or muscle cross-sectional area (Fig. 4*B*). Thus, neural input from the central nervous system could alter accompanying global changes, consisting of the whole body condition and nutrition, and the change in neural input might compensate for the changes in local muscle.

The blood sample test did not reveal pathologies as all data were within normal ranges (Table 1). For the liver function, total protein, AST, and ALT were within the standard ranges, suggesting that an excess catabolic process did not occur in this case (15). However, the total protein for WL was slightly lower than that for CON. This might suggest the cause of the decline in skeletal muscle mass for WL compared with CON. Focusing on the difference in glycerides between the two conditions, levels were lower for WL than CON from 3 to 1 wk before the matches; therefore, weight loss might be partly due to malnutrition, although the values were within standard ranges. Albumin, which is widely used as an index of malnutrition, was not significantly different between the conditions, but its level does not change in acute phases (16). Glucose levels were slightly elevated within the standard range; however, there was no marked difference between WL and CON. The acute weight loss for WL might not be explained by the state of the glucose level. Regarding the kidney function, there were no notable differences between WL and CON.

The present case study had some limitations. One of them was that we did not investigate caloric intake. Acute weight loss in combat athletes is primarily considered to be due to dehydration (17). A previous study reported that muscle glycogen, which is bound to abundant water and stored in muscle, decreased during weight loss in wrestlers (18). In the present case study, total body water decreased by ∼1%–2% from the baseline for WL (Fig. 2*D*). Skeletal muscle mass evaluated by bioimpedance also decreased by ∼1%–2% for WL (Fig. 2*B*), but mCSA decreased by ∼4% for WL (Fig. 3*B*). The decrease in muscle volume was mainly caused by dehydration, but this did not explain all losses, especially the decrease of local muscle mass, which could not be explained by dehydration alone. Unfortunately, we did not investigate the diet and caloric intake in this case study. In a future study, detailed dietary data should be collected. Another limitation was that we could not track the same motor units through the periods. Future studies should track the same motor units, which will provide more detailed physiological information.

In conclusion, this case study was a pilot, being the first study to investigate neuromuscular characteristics including the central nervous system, evaluated by HDsEMG with changes in body weight in an athlete. The findings suggest that body weight loss leads to an increase in the motor unit discharge rate to compensate for a decline in muscle mass and maintain muscle strength. Future studies should investigate the phenomenon in many athletes or nonathletes, such as patients who require increased neural input to exert muscle strength. For example, older adults show more trainability of neural than morphological factors (19, 20). It will be of interest to identify the association between body weight and neural factors in many subjects.

## GRANTS

This study was supported by a Grant-in-Aid from the Japan Society for the Promotion of Science Fellows (21J00674).

## DISCLOSURES

No conflicts of interest, financial or otherwise, are declared by the authors.

## AUTHOR CONTRIBUTIONS

T.H. and K.W. conceived and designed research; T.H. performed experiments; T.H., S.U., and Y.M. analyzed data; T.H. and K.W. interpreted results of experiments; T.H. prepared figures; T.H. drafted manuscript; T.H. and K.W. edited and revised manuscript; T.H., S.U., Y.M., and K.W. approved final version of manuscript.
